# HBeAg induces liver sinusoidal endothelial cell activation to promote intrahepatic CD8 T cell immunity and HBV clearance

**DOI:** 10.1038/s41423-021-00769-7

**Published:** 2021-09-15

**Authors:** Xiaohong Xie, Jinzhuo Luo, Ruth Broering, Dan Zhu, Wenqing Zhou, Mengji Lu, Xin Zheng, Ulf Dittmer, Dongliang Yang, Jia Liu

**Affiliations:** 1 Department of Infectious Diseases, Union Hospital, Tongji Medical College, Huazhong University of Science and Technology, Wuhan, Hubei China; 2 Department of Gastroenterology and Hepatology, University Hospital of Essen, University of Duisburg-Essen, 45122 Essen, Germany; 3 Institute for Virology, University Hospital of Essen, University of Duisburg-Essen, 45122 Essen, Germany

**Keywords:** Hepatitis B, Cytotoxic T cells

## To the Editor

It is believed that the inherent tolerogenic property of the liver is involved in the chronicity of hepatitis B virus (HBV) infection [1]. However, exposure to HBV in adults usually leads to spontaneous clearance of the virus and the induction of potent and effective anti-HBV T cell immunity in the liver [2], suggesting that the immune microenvironment of the liver switches from limiting to allowing effector T cell responses during acute resolution of HBV infection. To date, it remains largely unknown how the immune microenvironment of the liver is regulated and by which mechanism a favorable intrahepatic anti-HBV T cell response is generated in an infected individual. Liver sinusoidal endothelial cells (LSECs) play key roles in intrahepatic immune surveillance against infection by regulating the activation of local immune cells [3]. We have previously demonstrated that LSECs switch from a tolerogenic to an immunogenic state and trigger cytotoxic effector CD8 T cell activation under inflammatory conditions [4, 5]. Here, we investigated whether LSECs exhibit plasticity and switch from a tolerogenic to an immunogenic state upon HBV exposure.

**Fig. 1:**
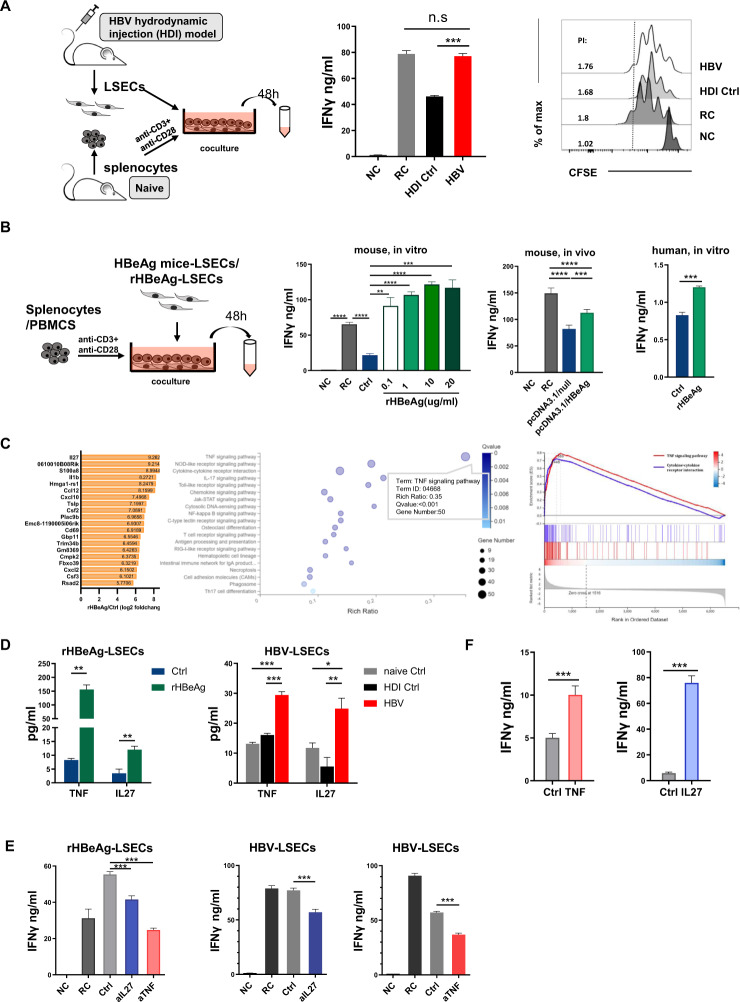
HBeAg induces liver sinusoidal endothelial cell activation to promote intrahepatic CD8 T cell immunity and HBV clearance. **A** LSECs from mice that were hydrodynamically injected with pSM2 plasmid (HBV) or PBS (HDI Ctrl) were isolated 14 days postinjection (dpi) and cocultured with polyclonally stimulated splenocytes at a ratio of 1:2 (LSECs: splenocytes). Anti-CD3/anti-CD28–stimulated splenocytes were used as a responder control (RC). Unstimulated splenocytes were used as a negative control (NC). IFNγ production was measured after 48 h. CFSE-labeled polyclonally stimulated splenocytes were cocultured with LSECs, and after 72 h, CD8 T cell proliferation was analyzed by flow cytometry. **B** Naïve mouse or primary human LSECs were treated with recombinant HBeAg (rHBeAg) or left untreated (Ctrl) for 24 h, washed and cocultured with polyclonally stimulated splenocytes or PBMCs at a ratio of 1:2. LSECs from mice that were hydrodynamically injected with pcDNA3.1/HBeAg or the pcDNA3.1/null plasmid at 2 dpi were isolated and cocultured with polyclonally stimulated splenocytes at a ratio of 1:2. IFNγ production was measured after 48 h. Anti-CD3/anti-CD28–stimulated splenocytes were used as a responder control (RC). Unstimulated splenocytes were used as a negative control (NC). **C** LSECs from naïve mice were mock-treated or treated with rHBeAg, and total RNA was extracted from the cells 6 h later for RNA-seq analysis. Log2-fold changes in the top 20 differentially expressed genes (DEGs), KEGG pathway enrichment analysis and gene set enrichment analysis (GSEA), as determined by RNA-seq analysis. **D** Supernatants of rHBeAg-LSECs or HBV-LSECs were analyzed for TNF α and IL-27. **E** Polyclonally stimulated splenocytes were cocultured with rHBeAg-LSECs or HBV-LSECs, and then 10 μg/ml anti–IL-27 Abs (aIL-27) or anti–TNFα Abs (aTNFα) were added to the cocultures, with untreated cocultures as the control (Ctrl). IFNγ production was measured by ELISA after 48 h. **F** Polyclonally stimulated splenocytes were cocultured with LSECs from naïve mice, and 50 ng/ml rIL-27 or 100 ng/ml rTNFα was added to the cocultures. IFNγ production was measured by ELISA after 48 h. Anti-CD3/anti-CD28-stimulated splenocytes only were used as a responder controls (RC). Unstimulated splenocytes were used as a negative control (NC). Unpaired *t*-test or one-way ANOVA is used. Error bars, mean ± SEM; *p < 0.05; **p < 0.01; ***p < 0.001.

We first analyzed LSEC-mediated T cell suppression during the course of acute resolution of HBV replication using the HBV hydrodynamic injection (HDI) mouse model. LSECs were freshly purified at 14 days post injection (dpi) and cocultured with T cell receptor (TCR)-activated T cells (Fig. 1A). These T cells produced significantly higher levels of IFNγ than those cocultured with control LSECs (Fig. 1A). LSECs from HBV HDI mice also showed less suppression of CD8 T cell proliferation than LSECs from control mice (Fig. 1A). These results suggest that the ability of LSECs to suppress T cell activation was abolished in HBV HDI mice at 14 dpi. Next, we examined whether exposure to specific HBV antigens altered the ability of LSECs to suppress T cell activation. Our data showed that in vitro pretreatment of LSECs with recombinant HBeAg (rHBeAg) completely abolished the suppression of T cell IFNγ production in a dose-dependent manner (Fig. 1B). Activated T cells cocultured with LSECs exposed to HBeAg in vivo also showed significantly increased IFNγ production compared with those cocultured with control LSECs (Fig. 1B). Moreover, CD3/CD28 Dynabead-activated human PBMCs cocultured with rHBeAg-pretreated human LSECs produced significantly higher amounts of IFNγ than PBMCs cultured with control LSECs (Fig. 1B). These data suggest that HBeAg stimulation could abrogate LSEC-mediated T cell suppression in both mice and humans.

To further examine the possible mechanisms by which HBeAg-exposed LSECs (HBeAg-LSECs) regulate T cell activation, HBeAg-LSECs and unstimulated control LSECs (Ctrl-LSECs) were subjected to transcriptome RNA sequencing analysis (Fig. S1A–C). The pathway functional enrichment of coexpressed DEGs analysis and gene set enrichment analysis (GSEA) revealed that gene sets associated with the cytokine–cytokine receptor interaction and TNF signaling pathway were enriched in HBeAg-LSECs (Fig. 1C), among which the most upregulated gene was IL27 (~613-fold increase), and the TNF signaling pathway exhibited the maximum enriched ratio (Figs. 1C and S1D). Collectively, these data suggest that HBeAg-LSECs induce T cell activation by producing cytokines. This result was further confirmed by the observation that the transfer of HBeAg-LSEC supernatants into cocultures of activated T cells and untreated LSECs significantly increased IFNγ production by activated T cells (Fig. S1E). Next, we examined the roles of TNF and IL27 in HBeAg-LSEC-induced T cell immunity. Significantly increased amounts of TNF and IL27 were measured in the supernatant of HBeAg-LSECs after 24 h of in vitro stimulation or LSECs purified from HBV HDI mice compared with those produced by the corresponding Ctrl-LSECs (Figs. 1D and S2A, B). TNF or IL27 blockade significantly abrogated IFNγ production by activated T cells cocultured with HBeAg-LSECs or LSECs from HBV HDI mice (Figs. 1E and S2C, D). In contrast, adding recombinant TNF or IL27 to cocultures of activated T cells and untreated LSECs significantly increased IFNγ production by activated T cells (Figs. 1F and S2E).

Taken together, our study highlights a previously unappreciated role of HBeAg in inducing LSECs to trigger specific T cell activation partially by increasing IL27 and TNF expression and provides a new regulatory mechanism of the intrahepatic immune microenvironment during HBV infection.

## Supplementary information

supplemental material
