# Supplementary material for: HBeAg induces liver sinusoidal endothelial cell activation to promote intrahepatic CD8 T cell immunity and HBV clearance
Source: Cell Mol Immunol. 2021 Sep 15;18(11):2572–4. doi: 10.1038/s41423-021-00769-7 (PMC8546047; doi:10.1038/s41423-021-00769-7)
Supplement: Supplementary file 1 — supplemental material [file 41423_2021_769_MOESM1_ESM.docx]

**HBeAg induces liver sinusoidal endothelial cell activation to promote intrahepatic CD8 T cell immunity and HBV clearance**

Xiaohong Xie^1 *^, Jinzhuo Luo^1*^, Ruth Broering^2^, Dan Zhu^1^, Wenqing Zhou^1^, Mengji Lu^3^, Xin Zheng^1^, Ulf Dittmer^3^, Dongliang Yang^1^, Jia Liu^1^

*^1^ Department of Infectious Diseases, Union Hospital, Tongji Medical College, Huazhong University of Science and Technology, Wuhan, Hubei, China*

*^2^ Department of Gastroenterology and Hepatology, University Hospital of Essen, University of Duisburg-Essen, 45122 Essen, Germany*

*^3^ Institute for Virology, University Hospital of Essen, University of Duisburg-Essen, 45122 Essen, Germany*

* These authors contributed equally to this article.

**Correspondence to:**

Prof. Dr. Jia Liu, e-mail: [jialiu77@hust.edu.cn](mailto:jialiu77@hotmail.com),

Tel: +8618696159826

Department of Infectious Diseases, Union Hospital, Tongji Medical College, Huazhong University of Science and Technology, Wuhan 430022, China

**Materials and Methods**

**Mice**

Male wildtype C57BL/6 mice were purchased from Hunan Slack King Laboratory Animal Co., Ltd. (Changsha, China). All animals were bred and kept under specific pathogen-free (SPF) conditions in the Animal Care Center of Tongji Medical College (Wuhan, China).

**Plasmids, antibodies and reagents**

The antibodies used in this study are listed in Supplementary Table 1. All plasmids were prepared using Endo-Free Plasmid Kits (Omega, Norcross, GA, USA). All of the above operations were performed according to the manufacturer’s instructions.

**Hydrodynamic injection in mice**

Hydrodynamic injection was performed as described previously by using plasmids to establish HBV replication in mice ^1^. In brief, male mice (6 to 8 weeks of age) were injected with 10 μg plasmids in a volume of phosphate buffer saline (PBS) equivalent to 0.1 mL/g of the mouse body weight through the tail vein within 5-8 seconds.

**Mouse cell isolation**

Isolation of mouse LSECs was performed as described previously with a cell purity over 95%^2^, and less than 0.5% of NK cells and DCs were found in the purified LSECs by using this isolation procedure^3^. Isolation of splenocytes and intrahepatic infiltrated lymphocytes was performed as described previously^4^. All isolated cell fractions contained less than 5% dead cells.

**Patient material and human LSEC culture experiment**

For validation purposes primary human LSEC were prepared from nontumor liver tissue obtained from fresh tumor resections as described previously^5^. All patients provided written documentation of informed consent. The study conforms to the ethical guidelines of the 1975 Declaration of Helsinki and was approved by the Institutional Review Board (Ethics Committee) of the medical faculty at the University Duisburg-Essen. Briefly, total liver cell suspension was obtained after two-step perfusion and digestion using collagenase IV. Parenchymal and nonparenchymal liver cells were separated by low-speed centrifugation steps and LSECs were prepared using CD146-based MACS bead separation kit (Miltenyi Biotec, Bergisch Gladbach, Germany). LSEC were seeded in collagen I coated 24-well plates and cultured in Endothelial Growth Medium 2 (PromoCell, Heidelberg, Germany) containing provided supplements, 100 U/ml penicillin, and 0.1 mg/ml streptomycin. After reaching 80% confluence LSEC were treated with rHBeAg (10 µg/ml) in RPMI supplemented with 10% FBS, 100 U/ml penicillin, 0.1 mg/ml streptomycin, and 2 mM L-glutamine for 24 h. Cells were washed three times with PBS and cocultured with 5×10^5 PBMC / 500 µl RPMI (peripheral blood mononuclear cell; healthy donor), preactivated with Dynabeads (Human T-Activator CD3/CD28, Invitrogen) according to manufactures instruction. Supernatants were collected after 48 h and IFNγ secretion was determined (Quantikine ELISA Kit, R&D, Minneapolis, MN, USA).

**Flow cytometry**

Cell surface and intracellular staining for flow cytometry analysis was performed as described previously ^4, 6^. The antibodies used for surface and intracellular staining are listed in Supplementary Table 2. Freshly isolated cells were used for all assays, and data were acquired using a FACS Canto II flow cytometer (BD Biosciences, San Jose, CA, USA) and analyzed using FlowJo software (Tree Star, Ashland, OR, USA). Cell debris and dead cells were excluded from the analysis based on scatter signals and Fixable Viability Dye eFluor 506 (eBioscience, San Jose, CA, USA).

**Cytokine assays**

LSECs were cultured 5×10^5 cells per well pre-stimulated with or without recombinant HBeAg (ProSpec, Ness-Ziona, Israel) in a total volume of 500μl, cell-free supernatants were collected and subjected to assays to measure cytokines using cytokine ELISA kits (eBioscience). Red blood cell-depleted splenocytes were cultured 1×10^6 cells per well with or without LSECs at a ratio of 2:1 (splenocytes to LSECs) in a total volume of 500μl. Splenocytes were stimulated with 1 μg/mL anti-CD3 and 1 μg/mL anti-CD28 (BD Bioscience). Cell-free supernatants were collected and subjected to assays to measure IFNγ, IL27 and TNF production using cytokine ELISA kits (eBioscience).

**Analysis of T cell proliferation and function**

Carboxyfluorescein succinimidyl ester (CFSE) (Invitrogen, Carlsbad, CA, USA)-labeled T cells were coincubated with LSECs for 3 days and analyzed for proliferation by flow cytometry.

**Statistical analysis**

Statistical analyses were performed using the SPSS statistical software package (version 22.0, SPSS Inc., Chicago, IL, USA). The Shapiro-Wilk method was used to test for normality. Parametric analysis methods were used when the data were normally distributed; otherwise, non-parametric tests were employed. Unpaired t test, paired t test, one-way ANOVA, Pearson product-moment correlation coefficient, log-rank test, and analysis of covariance (ANCOVA) were used where appropriate. All reported p values were two-sided, and a p value less than 0.05 was considered statistically significant.

**Supplementary figure**

**Figure S1.**


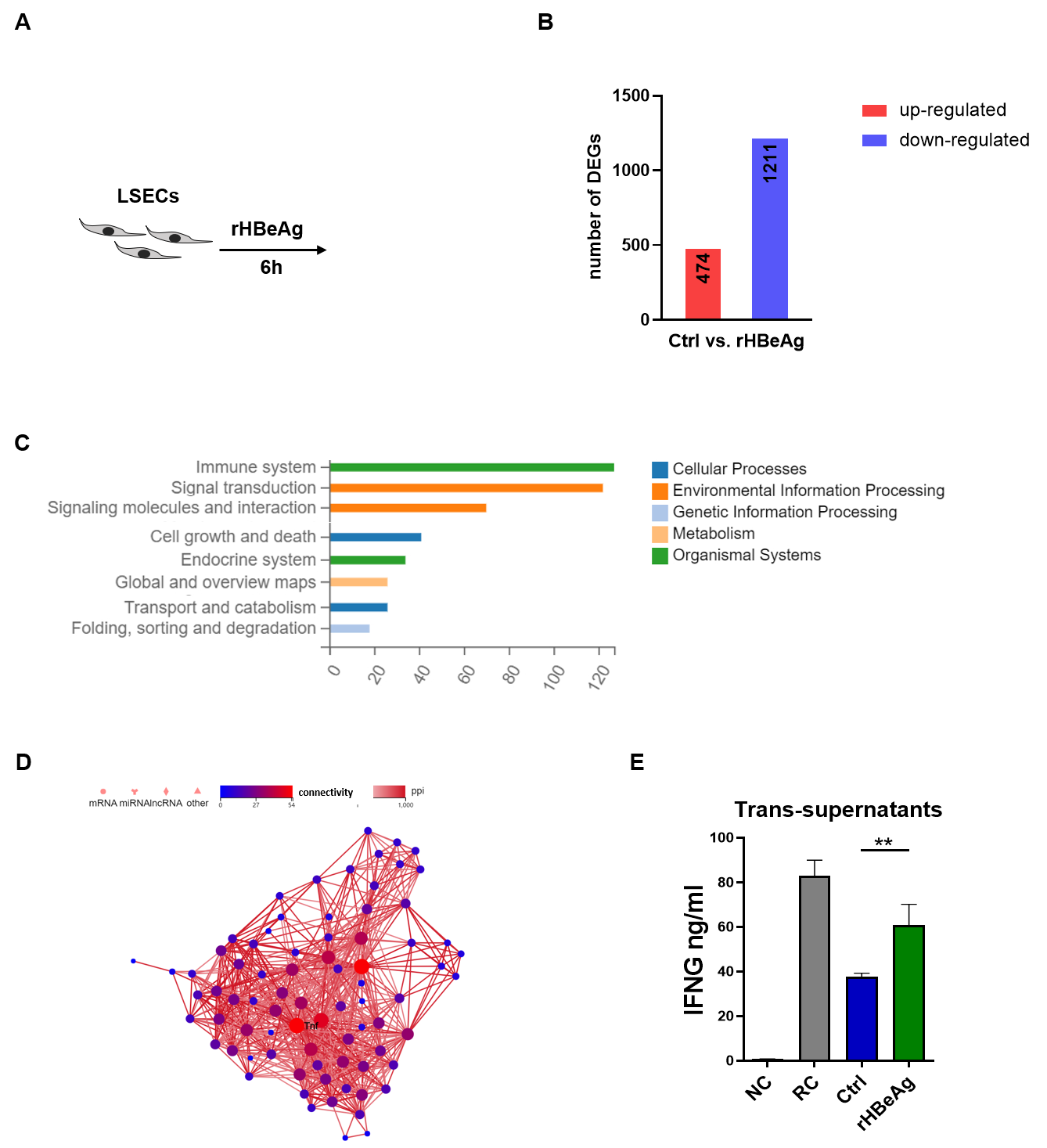


(A) LSECs from naïve mice were mock-treated or treated with rHBeAg, total RNA of the cells was extracted 6 h later for RNA-seq analysis. (B) Statistic of differentially expressed genes (DEGs). X axis represents pairwise and Y axis means number of screened DEGs. (C) KEGG analysis of the DEGs, top 3 significantly enriched parts of each domain were presented. (D) Protein–protein interaction (PPI) network of DEGs on TNF signaling pathway, the size of each node presents the degree of connectivity in PPI network, large node shares more connection to other nodes. (E) LSECs from naïve mice were incubated with 10 μg/ml rHBeAg or not (Ctrl) for 24 h. Supernatants of the LSECs was then harvested and transferred to the polyclonal stimulated splenocytes. IFNγ production by T cells was measured by ELISA after 48 h. Anti-CD3/anti-CD28-stimulated splenocytes only were used as responder controls (RC). Unstimulated splenocytes were used as negative control (NC). One-way ANOVA is used. Error bars, mean ± SEM; *p<0.05; **p<0.01; ***p<0.001.

**Figure S2.**


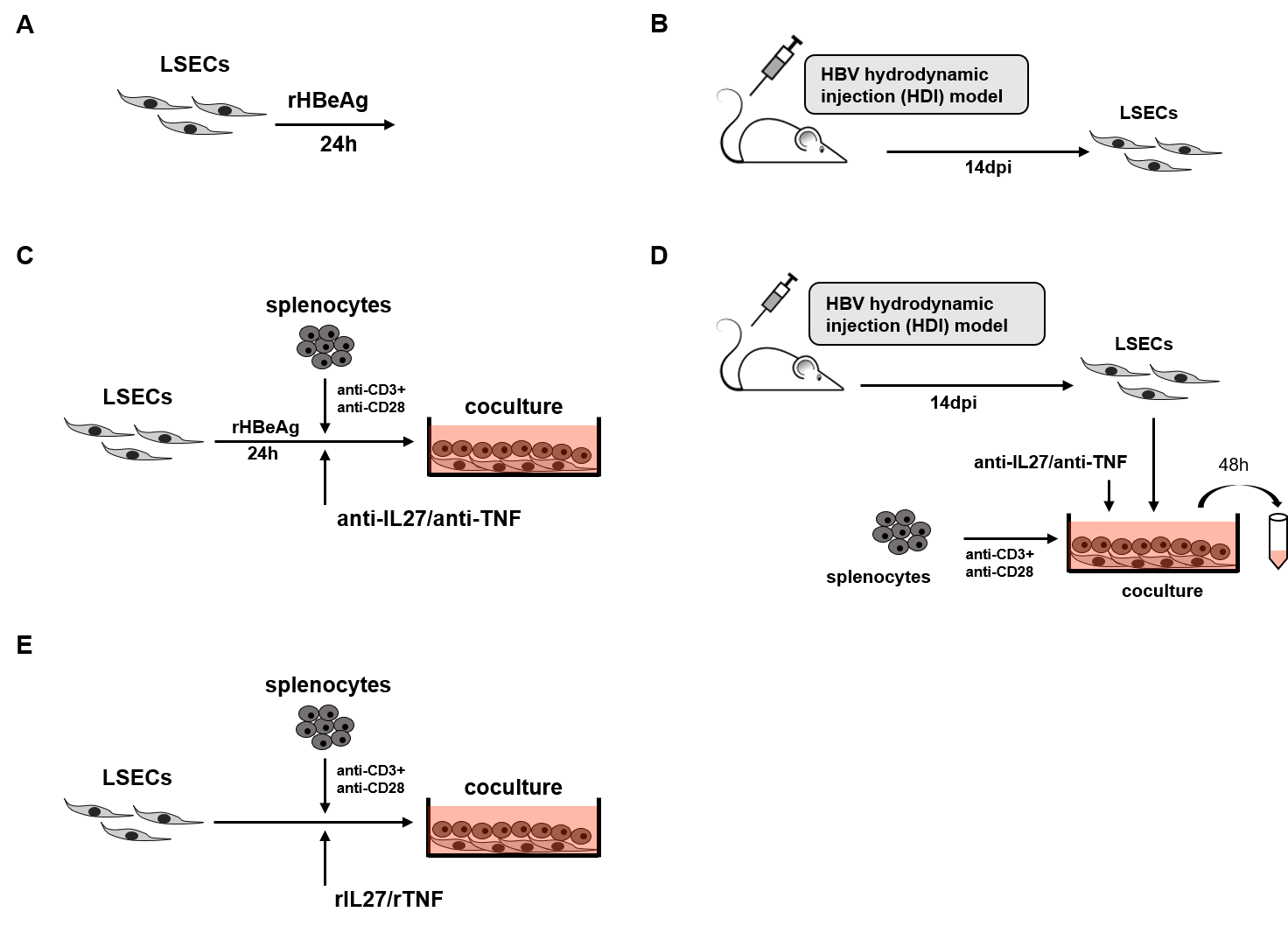


**Experimental scheme.** (A) LSECs from naïve mice were pretreated with 10 μg/ml rHBeAg for 24 h. Supernatant of LSECs was for TNF and IL27 detection. (B) LSECs from mice hydrodynamically injected with pSM2 plasmid (HBV) or PBS (HDI control) were separated at 14dpi and cultured for 24h. Supernatant of LSECs was detected for TNF and IL27. LSECs from naïve mice were used as naïve control. (C) Polyclonally stimulated splenocytes were cocultured with 10 μg/ml rHBeAg-pretreated LSECs, and 10 μg/ml anti–IL 27 Abs (aIL27) or anti–TNF Abs (aTNF) or not (Ctrl) were added to the cocultures. IFNγ production was measured by ELISA after 48 h. Anti-CD3/anti-CD28-stimulated splenocytes only were used as responder controls (RC). Unstimulated splenocytes were used as negative control (NC). (D) LSECs from mice hydrodynamically injected with pSM2 plasmid were separated at 14dpi and cocultured with polyclonal stimulated splenocytes at a ratio of 1:2 (LSECs: splenocytes). 10 μg/ml anti–IL27 Abs (aIL27) or anti–TNF Abs (aTNF) or not (Ctrl) were added to the cocultures. IFNγ production was measured by ELISA

after 48 h. Anti-CD3/anti-CD28–stimulated splenocytes only were used as responder controls (RC). Unstimulated splenocytes were used as negative control (NC). (E) Polyclonal stimulated splenocytes were cocultured with LSECs from naïve mice, and 50ng/ml rIL27 or 100ng/ml rTNF were added to the cocultures. IFNγ production was measured by ELISA after 48 h.


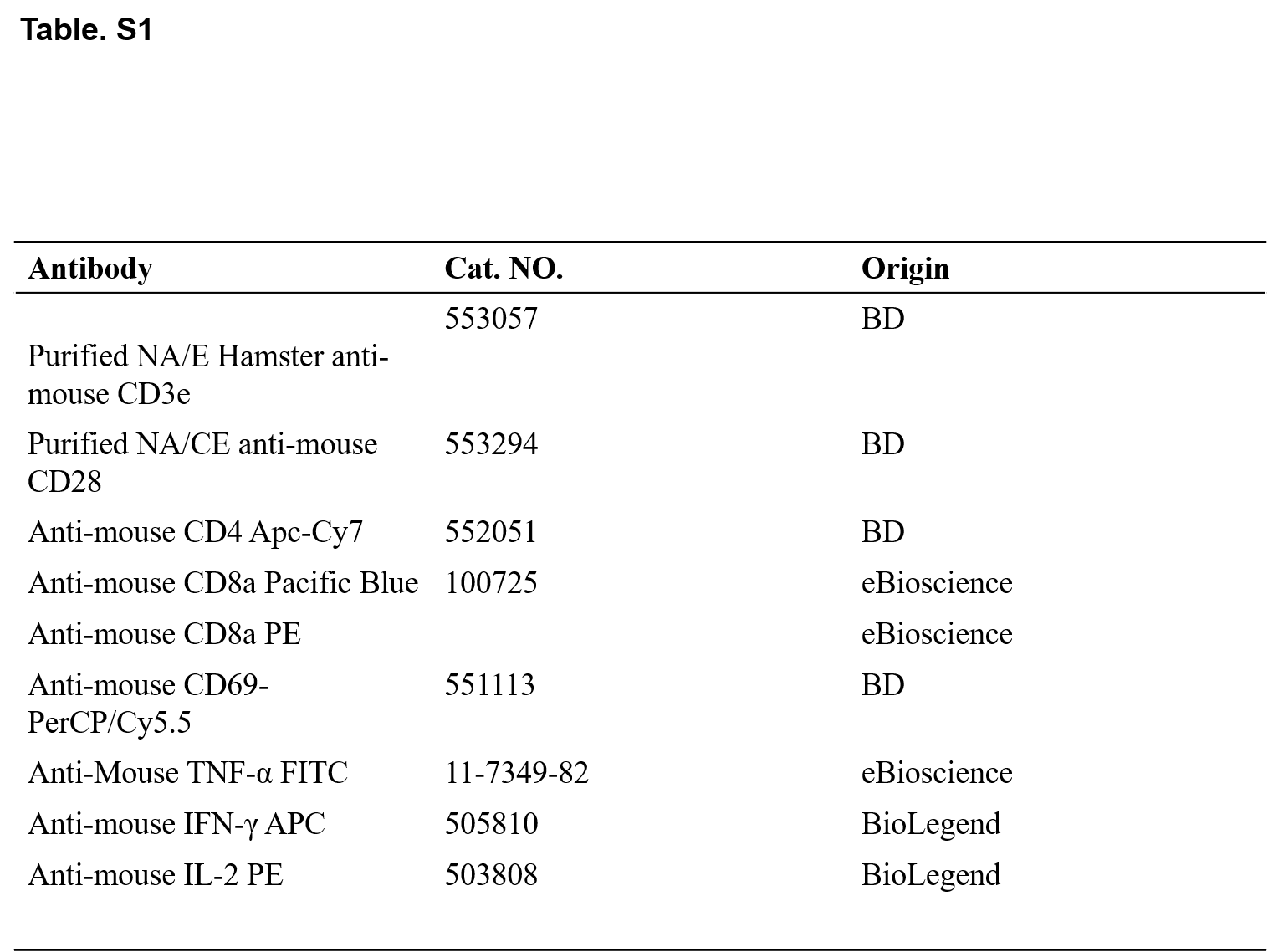


1. Wang Q, Pan W, Liu Y, Luo J, Zhu D, Lu Y, et al. Hepatitis B Virus-Specific CD8+ T Cells Maintain Functional Exhaustion after Antigen Reexposure in an Acute Activation Immune Environment. Frontiers in immunology. **9**, 219(2018)

2. Liu J, Huang X, Werner M, Broering R, Yang D, Lu M. Advanced Method for Isolation of Mouse Hepatocytes, Liver Sinusoidal Endothelial Cells, and Kupffer Cells. Methods in molecular biology (Clifton, NJ). **1540**, 249-58(2017)

3. Liu J, Jiang M, Ma Z, Dietze KK, Zelinskyy G, Yang D, et al. TLR1/2 ligand-stimulated mouse liver endothelial cells secrete IL-12 and trigger CD8+ T cell immunity in vitro. J Immunol. **191**, 6178-90(2013)

4. Yang S, Wang L, Pan W, Bayer W, Thoens C, Heim K, et al. MMP2/MMP9-mediated CD100 shedding is crucial for inducing intrahepatic anti-HBV CD8 T cell responses and HBV clearance. J Hepatol. **71**, 685-98(2019)

5. Werner M, Driftmann S, Kleinehr K, Kaiser GM, Mathé Z, Treckmann J-W, et al. All-In-One: Advanced preparation of Human Parenchymal and Non-Parenchymal Liver Cells. PloS one. **10**, e0138655(2015)

6. Liu J, Yu Q, Wu W, Huang X, Broering R, Werner M, et al. TLR2 Stimulation Strengthens Intrahepatic Myeloid-Derived Cell-Mediated T Cell Tolerance through Inducing Kupffer Cell Expansion and IL-10 Production. J Immunol. **200**, 2341-51(2018)
